# Intraspecific higher order interactions enhance ecological community stability

**DOI:** 10.1038/s41598-025-15320-1

**Published:** 2025-08-17

**Authors:** Akihiko Mougi

**Affiliations:** Institute of Agricultural and Life Sciences, Academic Assembly, Shimane University, 1060 Nishikawatsu-Cho, Matsue, 690–8504 Japan

**Keywords:** Higher-order interaction, Complexity–stability, Intraspecific interaction, Niche, Mathematical model, Ecology, Theoretical ecology

## Abstract

Ecosystem stability is influenced by interspecific interactions, with recent research focusing on how third-party species modify these interactions. However, the impact of "intraspecific higher-order interactions," where the presence of one species affects intraspecific interactions within another species, on ecosystem stability has been less explored. This study addresses this gap by developing a mathematical model to investigate how such interactions influence stability. The analysis shows that when higher-order interactions increase intraspecific competition within another species, stability improves, especially in large, complex ecosystems. However, this effect is not observed if the interactions solely increase or decrease competition without a mixture of both. These findings highlight the importance of both positive and negative effects on intraspecific competition for enhancing stability in complex ecosystems. This emphasizes the need for further research on the role of higher-order interactions in shaping ecosystem stability, particularly in complex ecosystems.

**Supplementary Information:**

The online version contains supplementary material available at 10.1038/s41598-025-15320-1.

## Introduction

Understanding how ecosystems maintain stability is a central challenge in ecology^1,3^. According to May’s theory, as the complexity of a community (species richness and the density of interspecific interactions) increases, the stability of the ecosystem tends to decrease^1^ (negative complexity-stability relationship). This contradicts the intuitive notion that greater diversity should enhance stability (positive complexity-stability relationship), as observed in natural ecosystems with diverse species. To address this paradox, traditional ecological research has primarily focused on direct interspecific interactions such as competition, predation, and mutualism and their effects on ecosystem dynamics and stability^4,17^. These studies have revealed how these interactions shape interaction network structure and population dynamics, providing insights into the factors that influence ecosystem stability^18^. However, a critical aspect that remains underexplored is the role of higher-order interactions or interaction modifications—situations where the presence of additional species alters the interactions between other species within a community— which may play a crucial role in influencing stability^19,28^. Addressing this gap in research could provide a more nuanced understanding of how complex ecosystems achieve stability despite the challenges highlighted by May’s theory^1^.

Despite the progress made in understanding these types of “interspecific” higher-order interactions, less attention has been paid to how these interactions might influence intraspecific interaction, the interaction among individuals within the same species. Recently studies are beginning to address this gap, showing that the presence of other species can alter intraspecific interaction through mechanisms such as non-consumptive effects^29,30^. For example, predators may increase intraspecific competition within prey species by causing individuals to seek shelter and thus compete more intensively for limited resources, or they may reduce intraspecific competition by dispersing prey and diminishing density-dependent effects^31,32^. Empirical studies also suggest that the removal of other species can lead to niche expansion and changes in competition dynamics^33^, further indicating that the presence/absence of other species influences intraspecific competition^34,36^. Additionally, species that do not interact directly may still impact community dynamics through environmental modifications, engineering roles that alter habitats and affect the niches and competition of other species^37^. Although the mechanisms may vary, the presence of other species can affect intraspecific competition by either constraining or expanding the niches available to other species.

A prominent example of complex ecological interactions is trait-mediated effects^38,40^, where the presence of a species influences the traits or behaviors of other species, which in turn impacts their interactions. While these trait-mediated effects can lead to indirect interactions, it is important to distinguish them from true higher-order interactions. It refers to cases where the effect of one species on another is directly modified by the presence of a third species and cannot be captured by pairwise interactions alone^41^. Therefore, although trait-mediated effects and indirect interactions can appear similar to higher-order interactions, they are conceptually and mathematically distinct.

Against this backdrop, this study aims to delve deeper into the underexplored realm of how “intraspecific” higher-order interactions, specifically those affecting intraspecific competition, impact ecosystem stability. Theoretical studies examining how higher-order interactions influencing intraspecific interactions affect community stability are extremely limited^24^, and to date, there are no studies addressing the complexity-stability relationship.

**Fig. 1:**
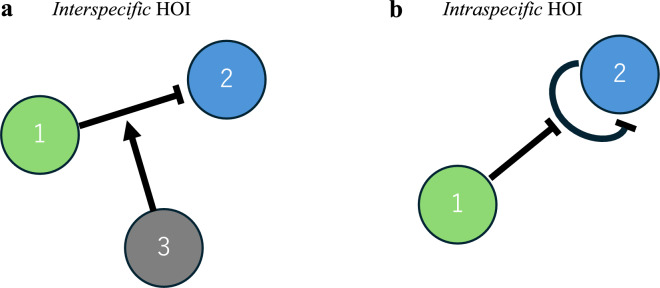
Higher-order interactions affecting interspecific (**a**) and intraspecific (**b**) interactions. Normal and hammerhead arrows represent positive and negative interaction effects. Circles represent different species. In the example of (**a**), species 3 modulates the interaction between species 1 and 2. In (**b**), species 1 modulates the intraspecific interactions within species 2.

Using a mathematical model, I construct random communities with various types of species interactions, where higher-order interactions affect the intraspecific competition of other species. It was assumed that these higher-order interactions could either strengthen (positive higher-order interaction) or weaken (negative higher-order interaction) intraspecific competition (Fig. 1). Note that positive higher-order interactions in this study is equivalent to negative higher-order interactions in earlier work^24^. Community stability is evaluated by local stability following May’s approach^1^ (Methods). Specifically, I hypothesize that positive higher-order interactions will enhance community stability, while negative higher-order interactions will reduce it. Additionally, the proportion of these interactions (*p*: proportions of positive higher-order interactions) may play a crucial role in determining the relationship between complexity and stability. The effects of higher-order interactions on stability are also expected to be independent of the type of community, whether it is a random community with various species interactions, a food web, a mutualistic community, or a competitive community.

## Results

**Fig. 2:**
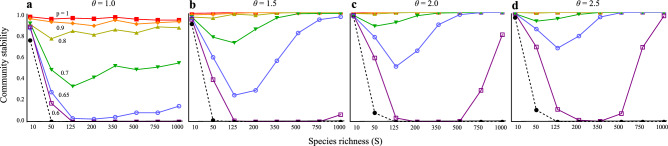
Relationships between species richness and community stability with varying the proportion of positive and negative HOI. (**a**–**d**) has different degrees of parameter variation. Higher *θ* means smaller variations of parameters among species. In an extreme (*θ* = 1), parameters are randomly chosen from uniform distribution *θ*. Black dashed lines are the results without HOIs (*h* = 0). Solid lines are the results with HOIs (*h* = 1). Colors in solid lines represent different values of *p* (proportion of positive higher-order interaction). I assume *C* (connectance) = 0.3.

**Fig. 3:**
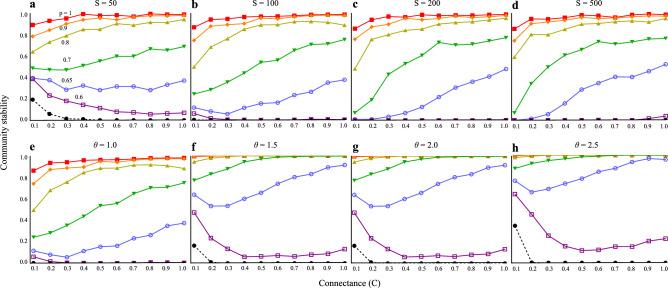
Relationships between connectance (*C*) and community stability with varying the proportion of positive HOI (*p*). (**a**–**d**) has different species richness (*S*). I assume *θ* = 1. (**e**–**h**) has different values of *θ* (variability). I assume *S* = 100. Black dashed lines are the results without HOIs (*h* = 0). Note that community stability is always zero in **b**–**e**. Solid lines are the results with HOIs (*h* = 1). Colors in solid lines represent different values of *p*.

In the absence of intraspecific higher-order interactions (*h* = 0), increased community complexity, species richness, and connectance generally destabilize communities (Figs. 2, 3). However, the introduction of higher-order interactions significantly alters this outcome.

When higher-order interactions are introduced (*h* = 1), the stability of communities is significantly enhanced, especially when the proportion of positive higher-order interactions is high (*p* > 0.8). In these cases, stability remains high and becomes less dependent on species richness (Fig. 2), with only a slight improvement in stability as connectance increases (Fig. 3). Communities with a higher proportion of positive higher-order interactions (higher values of *p* in Fig. 2) exhibit higher stability. Conversely, when the negative higher-order interactions increases (lower values of *p* in Fig. 2), community stability declines. To counteract the destabilizing effects of negative higher-order interactions, positive higher-order interactions must constitute more than 80% of the total interactions to maintain stability (Fig. 2). These findings support the hypothesis that negative higher-order interactions reduce stability, and the destabilization is mitigated by positive higher-order interactions.

A balance between positive and negative higher-order interactions, with a slight bias towards positive interactions, creates a distinct positive complexity-stability relationship. Across all parameter variation among species, an excess of negative interactions beyond 40% results in a negative complexity-stability effect, where increases in species richness and connectance reduce stability (Figs. 2, 3). However, this relationship improves as the parameter variation is reduced (e.g., in Fig. 2, moving from *a* to* b*). With a balanced mixture of different types of higher-order interactions, slightly biased towards positive interactions (0.6 ≤ *p* ≤ 0.7), species richness can have a non-monotonic effect on stability: initially decreasing stability, but eventually leading to recovery with further increases in richness (Fig. 2b-d). In contrast, connectance consistently induces a monotonic positive complexity-stability relationship, irrespective of parameter variation (Fig. 3), under the scenario when *p* > 0.65. These results indicate that the balance of positive and negative higher-order interactions plays a crucial role in determining the relationship between complexity and stability.

**Fig. 4:**
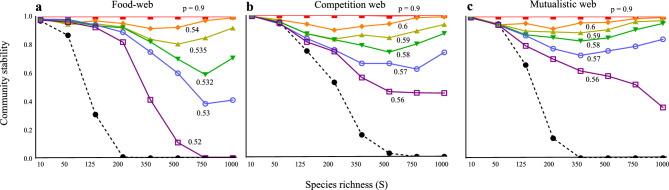
Complexity-stability relationship in different types of network with varying the proportion of positive HOI (*p*). (**a**) Food-web. (**b**) Competition web. (**c**) Mutualistic web. In each interaction web, random network is assumed. In food-web, symmetric interaction is assumed (i.e. conversion efficiency is 1). In competition and mutualistic webs, (c_*A*_, c_*B*_, c_*s*_) = (0.01, 0.1, 1.5). c_k_ indicates the control parameter that determines of the absolute values of each parameter (k = *A*, *B*, or *s*; where *A* is the interaction coefficient between species, *B* is the higher-order interaction coefficient between species, and *s* is the density-dependent self-regulation of each species). In food web, the parameter settings are same with the main model. Black dashed lines are the results without HOIs (*h* = 0). Solid lines are the results with HOIs (*h* = 1). Colors in solid lines represent different values of *p*. I assume *C* (connectance) = 0.3.

Furthermore, the community stability trends are robust across different types of interactions. The main analysis used random networks with various interaction types, and similar positive complexity-stability relationships were observed even in communities characterized by a single interaction type (e.g., food webs, competitive webs, mutualistic webs) when a moderate mixture of higher-order interactions was present (Fig. 4, Fig. S1). Additionally, the main analysis assumed that higher-order interactions occur “only” among interacting species pairs. However, higher-order interactions can occur independently of direct species interactions (e.g. ecosystem engineers can influence intraspecific competition of other non-interacting species by either narrowing or expanding a niche). This difference in assumptions had negligible effect on the results: (i) stabilizing effect of positive higher-order interactions and (ii) a positive complexity-stability relationship emerged by a balanced mixture of different types of higher-order interactions (Fig. S2). The positive complexity-stability relationship can be also observed even with relatively weak effects of higher-order interactions compared with pairwise direct interactions (*h* < 1) (Fig. S3). As *h* decreases, the level of complexity (species richness) required to increase stability becomes higher.

## Discussion

This study provides new insights into how intraspecific higher-order interactions influence ecosystem stability, with a particular focus on how these interactions shape the complexity-stability relationship. The results support the hypothesis that positive intraspecific higher-order interactions enhance stability, while negative intraspecific higher-order interactions decrease it. The stabilization effect of higher-order interactions on intraspecific competition is not surprising. In communities dominated by positive higher-order interactions, self-regulation through intraspecific competition can outweigh interspecific interactions, leading to enhanced stability. This finding aligns with a previous study, which suggests that stability arises when interspecific interactions are weaker than self-regulation mechanisms^42^.

The ratio of positive to negative higher-order interactions has a decisive influence on the relationship between complexity and stability. Mixing positive and negative higher-order interactions, with a slight predominance of positive interactions, creates a positive complexity-stability relationship. Mathematical analysis supports the positive impact of complexity on stability due to a mixture of higher-order interactions (see SI Appendix). This analysis, which assumes parameter homogeneity and large system approximation^1,2^, shows that the system can exhibit: (i) Always unstable (without depending on complexity) if different types of higher-order interactions occur with similar frequencies (*p* $\approx$ 0.5) (Fig. S4); (ii) A positive complexity-stability relationship (Fig. S4) if the proportion of positive higher-order interactions is below a threshold ($p<\frac{1}{2}\left(1+\frac{1}{\sqrt{1+4{s}{\prime}}}\right)$, where *s*’ is self-regulation normalized by the mean interaction strength (*s*/*A*); and (iii) Always stable (without depending on complexity) (Fig. S4) if the proportion of positive higher-order interactions exceeds the threshold ($p>\frac{1}{2}\left(1+\frac{1}{\sqrt{1+4{s}{\prime}}}\right)$). The results approach these theoretical findings more closely as parameter variance decreases, although the general trend persists regardless of variance. This is of critical importance in the complexity-stability issue. The positive complexity-stability relationship due to higher-order interactions is not straightforward. In smaller communities where the number of species is low and such interactions are few, there is a possibility that some species may be biased towards negative higher-order interactions, even though the proportion of positive higher-order interactions may be greater than that of negative ones overall. In such cases, instability caused by negative higher-order interactions cannot be counterbalanced by positive higher-order interactions. Conversely, in larger communities, the situation can change significantly. Each species is influenced by a greater number of higher-order interactions, reducing the likelihood of any particular species being biased towards negative higher-order interactions. As a result, positive higher-order interactions must be sufficiently numerous to outweigh negative ones for each species, thereby enhancing stability. Although empirical studies remain limited, intraspecific higher-order interactions have been shown to exert substantial effects, both positive and negative^22,36^. Even in relatively simple systems involving only two or three species, these interactions can be as important as, or even more important than, direct interactions, contributing significantly to species persistence and community stability^36^. In contrast, interspecific higher-order interactions have not consistently shown a positive effect on the complexity–stability relationship. Prior theoretical work using random community models suggested that interspecific HOIs can constrain stability, especially in very small or very large communities^20^. However, intraspecific HOIs, particularly when stronger than interspecific ones^24^, can enhance stability even in more complex communities. Moreover, such stabilizing effects may arise even in the absence of direct pairwise interactions, for example, through ecosystem engineering effects, where species indirectly influence each other by modifying their shared environment^37^. Future research should aim to detect both intra- and interspecific HOIs in more diverse and larger ecological systems, and to assess their relative contributions to ecological stability.

The study also demonstrated that the effects of intraspecific higher-order interactions on complexity-stability relationship are not restricted to specific community types. Whether the community is a random network, a food web, a mutualistic system, or a competitive environment, higher-order interactions can stabilize the system. This finding is consistent with previous studies (e.g. 24). A theoretical study demonstrated that in competitive systems, multiple competitors can coexist if the higher-order interactions affecting intraspecific competition are stronger than those affecting interspecific competition^24^. The present study goes beyond this by demonstrating that intraspecific higher-order interactions become particularly important in more complex systems. However, to date, there are very few studies—regardless of methodology—that explicitly incorporate intraspecific higher-order interactions. In contrast, most existing studies have focused on interspecific higher-order interactions, often finding that they do not enhance community stability, particularly in systems with non-random trophic structures^43^. Some studies suggest that interspecific higher-order interactions may only contribute under specific, limited conditions—for instance, when higher-order interactions are weak and exhibit strong correlation with pairwise interactions in systems involving competition and facilitation^44^. Unlike interspecific higher-order interactions, the effects of intraspecific higher-order interactions appear to generalize across various types of interaction networks. Provided that their effects are not too weak relative to pairwise direct interactions, intraspecific higher-order interactions are likely to exert increasingly significant stabilizing influences as community complexity grows (Fig. S3). If the influence of interspecific higher-order interactions is indeed weak^44^, then the role of intraspecific higher-order interactions in stabilizing complex ecological systems becomes even more critical.

Finally, the theory offers important insights for biological conservation. Species extinctions or reductions in abundance can lead to a decrease in intraspecific higher-order interactions, potentially destabilizing the community. This destabilization may, in turn, further diminish the higher-order interactions, creating a feedback loop that exacerbates instability.

## Methods

Here, using a mathematical ecological community model, I explore how higher-order interactions affecting intraspecific competition of other species affect community stability.

By applying an extended definition of earlier studies on higher-order interactions (where the interaction between any two species is altered by a third species (Fig. 1a)), I define it in the present context as a change in interactions within a species caused by other species, regardless of their trophic position (Fig. 1b). The higher-order interactions either increase (positive higher-order interaction) or decrease (negative higher-order interaction) intraspecific competition of other species.

The community network is assumed to be random (with multiple interaction types including predation, competition and mutualism) or structured into food webs (with only predator–prey interaction), competition webs (with only competitive interaction) and mutualistic webs (with only mutualistic interaction). For a random community with *N* species coexisting, a pair of species *i* and *j* (*i*,* j* = 1,…, *N*) is connected by any interaction with probability *C* (connectance), defined as the proportion of realized interaction links (*L*) to the maximum possible interaction links (*L*_*max*_) in a given network model (*L* = *CL*_*max*_). *L*_max_ is calculated as *N*(*N* − 1)/2.

The community model is defined by an ordinary differential equation:

1 $$dX_{i} /dt = \left\{ {r_{i} + \Sigma_{j} A_{ij} X_{j} - (s_{i} + h\Sigma_{j} B_{ij} X_{j} )X_{i} } \right\}X_{i} ,$$

where *X*_*i*_ is the abundance of species *i*, *r*_*i*_ is the intrinsic rate of change for species *i*, *s*_*i*_ is the density-dependent self-regulation (strength of intraspecific competition) of species *i*, *A*_*ij*_ is the interaction coefficient between species *i* and *j* (*j* ≠ *i*), and *B*_*ij*_ is the higher-order interaction coefficient between species *i* and *j* (*j* ≠ *i*). Higher-order interactions affect species *i* through species *j*. Note that *s*_*i*_ and *B*_*ij*_ can also be written as *B*_*ii*_ and *B*_*iij*_, respectively^20^. The parameter *h* represents the strength of higher-order interactions and indicates the presence (*h* = 1) or absence (*h* = 0) of higher-order interactions in the main text. I assume that the higher-order interactions only occur among pairs of interacting species, regardless of their trophic levels, in the main text (this is relaxed in Fig. S2). The proportions of positive and negative higher-order interactions are *p* and 1 – *p*, respectively.

Parameters (*A*_*ij*_, *B*_*ij*_, *s*_*i*_, and* X*_i_* (equilibrium abundances)) are randomly selected from a beta-distribution c_k_β(*θ*, *θ*), where* θ* indicates variability in the distribution (assumed to be symmetric) and c_k_ is the control parameter that sets the absolute values of each parameter (k = *A*, *B*, *s* or X*). In main text, c_*A*_ = c_*B*_ = 0.1, c_*s*_ = 0.5 and c_X*_ = 1. The intrinsic rate of change *r*_*i*_ is determined such that *dX*_*i*_/*dt* = 0 holds after setting an equilibrium density *X*_i_* (> 0) for each species, *X*_i_*^45^. This means that all species are assumed to be feasible.

Stability analysis is based on the Jacobian community matrix^2^. The dynamics of small deviations *x* = (*x*_1_, *x*_2_,…, *x*_N_)^T^ from the equilibrium point, *X*_i_^*^ are given by:

$$dx/dt = Jx,$$

where *J* is the Jacobian matrix. Stability is defined as the probability of local equilibrium stability, estimated as the frequency of locally stable systems across 1000 sample communities. For each simulation, community networks are randomly constructed, and parameters are randomly determined by the above procedures. If the real part of the dominant eigenvalue of *J* is negative, the system is considered stable.

Additionally, I directly calculated the Jacobian matrix following May’s approach^2^, and the mathematical analysis supports the stabilization effect of higher-order interactions (Supporting Information).

## Supplementary Information

Supplementary Information.

## Data Availability

The datasets used and/or analysed during the current study available from the corresponding author on reasonable request.

## References

[1] May, R. M. Will a large complex system be stable?. *Nature* **238**, 413–414 (1972). 4559589 10.1038/238413a0

[2] Allesina, S. & Tang, S. The stability–complexity relationship at age 40: a random matrix perspective. *Popul. Ecol.* **57**(1), 63–75 (2015).

[3] McCann, K. S. The diversity–stability debate. *Nature* **405**(6783), 228–233 (2000). 10821283 10.1038/35012234

[4] Kondoh, M. Foraging adaptation and the relationship between food-web complexity and stability. *Science* **299**(5611), 1388–1391 (2003). 12610303 10.1126/science.1079154

[5] Okuyama, T. & Holland, J. N. Network structural properties mediate the stability of mutualistic communities. *Ecol. Lett.* **11**(3), 208–216 (2008). 18070101 10.1111/j.1461-0248.2007.01137.x

[6] Fowler, M. S. Increasing community size and connectance can increase stability in competitive communities. *J. Theor. Biol.* **258**(2), 179–188 (2009). 19490878 10.1016/j.jtbi.2009.01.010

[7] Melián, C. J., Bascompte, J., Jordano, P. & Krivan, V. Diversity in a complex ecological network with two interaction types. *Oikos* **118**(1), 122–130 (2009).

[8] Thébault, E. & Fontaine, C. Stability of ecological communities and the architecture of mutualistic and trophic networks. *Science* **329**(5993), 853–856 (2010). 20705861 10.1126/science.1188321

[9] Allesina, S. & Levine, J. M. A competitive network theory of species diversity. *Proc. Natl. Acad. Sci.* **108**(14), 5638–5642 (2011). 21415368 10.1073/pnas.1014428108 PMC3078357

[10] Mougi, A. & Kondoh, M. Diversity of interaction types and ecological community stability. *Science* **337**(6092), 349–351 (2012). 22822151 10.1126/science.1220529

[11] Coyte, K. Z., Schluter, J. & Foster, K. R. The ecology of the microbiome: networks, competition, and stability. *Science* **350**(6261), 663–666 (2015). 26542567 10.1126/science.aad2602

[12] Gellner, G. & McCann, K. Consistent role of weak and strong interactions in highand low-diversity trophic food webs. *Nat. Commun.* **7**, 11180 (2016). 27068000 10.1038/ncomms11180 PMC4832055

[13] Mougi, A. The roles of amensalistic and commensalistic interactions in large ecological network stability. *Sci. Rep.* **6**(1), 29929 (2016). 27406267 10.1038/srep29929 PMC4942820

[14] Mougi, A. & Kondoh, M. Food-web complexity, meta-community complexity and community stability. *Sci. Rep.* **6**, 24478 (2016). 27071716 10.1038/srep24478 PMC4829910

[15] Kawatsu, K. & Kondoh, M. Density-dependent interspecific interactions and the complexity–stability relationship. *Proc. Royal Soc. B Biol. Sci.* **285**(1879), 20180698 (2018). 10.1098/rspb.2018.0698 PMC5998089 29794052

[16] Mougi, A. Diversity of biological rhythm and food web stability. *Biol. Let.* **17**(2), 20200673 (2021). 33563135 10.1098/rsbl.2020.0673 PMC8086954

[17] Mougi, A. Dual species interaction and ecological community stability. *Ecology* **105**, e4251 (2024). 38272678 10.1002/ecy.4251

[18] Landi, P., Minoarivelo, H. O., Brännström, Å., Hui, C. & Dieckmann, U. Complexity and stability of ecological networks: A review of the theory. *Popul. Ecol.* **60**, 319–345 (2018).

[19] Abrams, P. A. Arguments in favor of higher order interactions. *Am. Nat.* **121**(6), 887–891 (1983).

[20] Bairey, E., Kelsic, E. D. & Kishony, R. High-order species interactions shape ecosystem diversity. *Nat. Commun.* **7**(1), 12285 (2016). 27481625 10.1038/ncomms12285 PMC4974637

[21] Grilli, J., Barabás, G., Michalska-Smith, M. J. & Allesina, S. Higher-order interactions stabilize dynamics in competitive network models. *Nature* **548**(7666), 210–213 (2017). 28746307 10.1038/nature23273

[22] Mayfield, M. M. & Stouffer, D. B. Higher-order interactions capture unexplained complexity in diverse communities. *Nat. Ecol. Evol.* **1**(3), 0062 (2017). 10.1038/s41559-016-0062 28812740

[23] Letten, A. D. & Stouffer, D. B. The mechanistic basis for higher-order interactions and non-additivity in competitive communities. *Ecol. Lett.* **22**(3), 423–436 (2019). 30675983 10.1111/ele.13211

[24] Singh, P. & Baruah, G. Higher order interactions and species coexistence. *Thyroid Res.* **14**(1), 71–83 (2021).

[25] Gibbs, T., Levin, S. A. & Levine, J. M. Coexistence in diverse communities with higher-order interactions. *Proc. Natl. Acad. Sci.* **119**(43), e2205063119 (2022). 36252042 10.1073/pnas.2205063119 PMC9618036

[26] Wilson, D. S. Complex interactions in metacommunities, with implications for biodiversity and higher levels of selection. *Ecology* **73**(6), 1984–2000 (1992).

[27] Billick, I. & Case, T. J. Higher order interactions in ecological communities: What are they and how can they be detected?. *Ecology* **75**(6), 1529–1543 (1994).

[28] Levine, J. M., Bascompte, J., Adler, P. B. & Allesina, S. Beyond pairwise mechanisms of species coexistence in complex communities. *Nature* **546**(7656), 56–64 (2017). 28569813 10.1038/nature22898

[29] Mitchell, M. D. & Harborne, A. R. Non-consumptive effects in fish predator–prey interactions on coral reefs. *Coral Reefs* **39**(4), 867–884 (2020).

[30] Wirsing, A. J., Heithaus, M. R., Brown, J. S., Kotler, B. P. & Schmitz, O. J. The context dependence of non-consumptive predator effects. *Ecol. Lett.* **24**(1), 113–129 (2021). 32990363 10.1111/ele.13614

[31] Sih, A. & Wooster, D. E. Prey behavior, prey dispersal, and predator impacts on stream prey. *Ecology* **75**(5), 1199–1207 (1994).

[32] Orrock, J. L. et al. Predator effects in predator-free space: the remote effects of predators on prey. *The Open Ecol. J.* **3**, 22–30 (2010).

[33] Bolnick, D. I. et al. Ecological release from interspecific competition leads to decoupled changes in population and individual niche width. *Proc. Royal Soc. B Biol. Sci.* **277**(1689), 1789–1797 (2010). 10.1098/rspb.2010.0018 PMC2871882 20164100

[34] Persson, L. Predator-mediated competition in prey refuges: the importance of habitat dependent prey resources. *Oikos* **68**, 12–22 (1993).

[35] Resetarits, W. J. Jr., Bohenek, J. R. & Pintar, M. R. Predator-specific responses and emergent multi-predator effects on oviposition site choice in grey treefrogs, Hyla chrysoscelis. *Proc. R. Soc. B* **288**(1950), 20210558 (2021). 33975473 10.1098/rspb.2021.0558 PMC8113890

[36] Shen, C., Lemmen, K., Alexander, J. & Pennekamp, F. Connecting higher-order interactions with ecological stability in experimental aquatic food webs. *Ecol. Evol.* **13**(9), e10502 (2023). 37693938 10.1002/ece3.10502 PMC10483096

[37] Jones, C. G., Lawton, J. H. & Shachak, M. Positive and negative effects of organisms as physical ecosystem engineers. *Ecology* **78**(7), 1946–1957 (1997).

[38] Beckerman, A. P., Uriarte, M. & Schmitz, O. J. Experimental evidence for a behavior-mediated trophic cascade in a terrestrial food chain. *Proc. Natl. Acad. Sci.* **94**(20), 10735–10738 (1997). 11038581 10.1073/pnas.94.20.10735 PMC23467

[39] Werner, E. E. & Peacor, S. D. A review of trait-mediated indirect interactions in ecological communities. *Ecology* **84**(5), 1083–1100 (2003).

[40] Holt, R. D., & Barfield, M. Trait-mediated effects, density dependence and the dynamic stability of ecological systems. *Trait-Med. Indirect Int.: Ecol. Evol. Perspect.* *89* (2012).

[41] Schmitz, O. J., Hambäck, P. A. & Beckerman, A. P. Trophic cascades in terrestrial systems: A review of the effects of carnivores on plants. *Am. Nat.* **155**(2), 141–153 (2000). 10686157 10.1086/303311

[42] Barabás, G., Michalska-Smith, J. M. & Allesina, S. The effect of intra-and interspecific competition on coexistence in multispecies communities. *Am. Nat.* **188**(1), E1–E12 (2016). 27322128 10.1086/686901

[43] Terry, J. C. D., Bonsall, M. B. & Morris, R. J. The impact of structured higher-order interactions on ecological network stability. *Thyroid Res.* **18**, 9 (2025).40001245

[44] Gibbs, T. L. et al. When can higher-order interactions produce stable coexistence?. *Ecol. Lett.* **27**, e14458 (2024). 38877741 10.1111/ele.14458

[45] Chen, X. & Cohen, J. E. Transient dynamics and food–web complexity in the Lotka-Volterra cascade model. *Proc. Royal Soc. Series B Biol. Sci.* **268**(1469), 869–877 (2001). 10.1098/rspb.2001.1596 PMC1088682 11345334

