# Supplementary material for: Intraspecific higher order interactions enhance ecological community stability
Source: Sci Rep. 2025 Aug 17;15:30074. doi: 10.1038/s41598-025-15320-1 (PMC12358509; doi:10.1038/s41598-025-15320-1)

Supplementary Information

Intraspecific higher order interactions and ecological community stability

Akihiko Mougi

SI text (Appendix)

Local stability analysis:

In Eq. (1) in the text, at the equilibrium, it holds that:

$$r_i = -\Sigma_j A_{ij} X_j^* + (s_i + h\Sigma_j B_{ij} X_j^*) X_i^* \quad (1)$$

The Jacobian matrix, J, is the linearization of Eq. (1) in the text at an equilibrium point with elements:

$$J_{ij} = \left. \frac{\partial(dX_i/dt)}{\partial X_j} \right|_{X^*} \quad (2)$$

The system is locally stable if all eigenvalues of J have negative real parts. For a random community matrix with $CN \gg 1$, $E(J_{ij}) = 0$ and $E(J_{ij}J_{ji}) = 0$, the stability criterion is given by (1, 2):

$$\sqrt{N \cdot Var(J_{ij})} < -J_{ii} \quad (3)$$

To ensure the above conditions were met, I applied some constraints to the community dynamics model. First, parameters and species abundances are set as constant ($s_i = s$, $A_{ij} = A$ and $X_i^* = X^*$). $B_{ij} = A$ or $-A$ and each proportion is p and $(1 - p)$, respectively. Then, parameters are set to be $X^* = 1/h(2p - 1)$ to hold that $E(J_{ij}) = 0$. Here, $p > 1/2$ is required for a feasible condition, $X^* > 0$.

As any pair of species is randomly connected with probability C in the model; there is no statistical correlation between the off-diagonal elements of the J matrix ($E(J_{ij}J_{ji}) = 0$). Therefore, for large systems, the diagonal elements J_{ii} and $\mathrm{Var}(J_{ij})$ ($= E(J_{ij}^2) - E(J_{ij})^2$) can be approximated by:

$$J_{ii} = -sX^* - C(N-1)hX^*(pAX^* - (1-p)AX^*) \quad (4a)$$

$$\mathrm{Var}(J_{ij}) = \{(N-1)C/N\}\{pX^{*2}A^2(1-hX^*)^2 + (1-p)X^{*2}A^2(1+hX^*)^2\} \quad (4b)$$

Then, substituting Eqs. (4) into Ineq. (3), I obtain the stability criterion (using $N \approx N-1$ and $X^* = 1/h(2p-1)$:

$$\sqrt{\frac{1}{(1-2p)^2} - 1} < \frac{s}{A\sqrt{CN}} + \sqrt{CN} \quad (5)$$

This represents two major things. First, the stability condition becomes stricter as p approaches to 0.5. Second, an increase of complexity (C and/or N) always contributes to stability. In addition, it can explain the positive complexity–stability relationship in a large value of p. Then, in smaller values of N, the condition (5) is likely to be met because the first term of the r.h.s. is very large. In a middle level of N, the condition (5) is not likely to be met because the first term of the r.h.s. becomes smaller and the second term of the r.h.s. is not large. However, a further increase of N increases the r.h.s., enabling stability condition to be met.

The stability criterion has other forms. First, it is arranged as a stability condition for p:

$$p > \hat{p}, \tag{6}$$

where $\hat{p} = \frac{1}{2} + \frac{1}{\sqrt{1+M+\frac{s}{A}(2+\frac{s}{AM})}}$ is the threshold of p for stability and $M = CN$ (hereafter referred to as complexity). The threshold increases within a range $M < s/A$, otherwise it decreases (if $M > s/A$). At very large M, the last term of the threshold approaches to zero, and $\hat{p} \approx 0.5$.

Second, the criterion (5) is arranged as a stability condition for M. The conditions are:

$$0 < M < \widehat{M}_l, \tag{7a}$$

$$\widehat{M}_u < M, \tag{7b}$$

where $\widehat{M}_l = \frac{1}{2}\left(L - \sqrt{L^2 - \frac{4s^2}{A^2}}\right)$ and $\widehat{M}_u = \frac{1}{2}\left(L + \sqrt{L^2 - \frac{4s^2}{A^2}}\right)$ (where $L = -1 + \frac{1}{(1-2p)^2} - \frac{2s}{A}$), which represents the lower and upper thresholds of M for stability, respectively. The conditions (7) indicates that in lower and higher complexity, the system can be stable but in a middle level of complexity ($\widehat{M}_l < M < \widehat{M}_u$), it can be unstable. This supports the finding of positive complexity-stability relationship in this paper. In a limit where $p \approx 0.5$, $\widehat{M}_l \approx 0$ and $\widehat{M}_u \approx \infty$. The proof is as follows. Let us assume that $p = 0.5 + \varepsilon$, where ε is a small value. In this case, the expression $\frac{1}{(1-2p)^2}$ simplifies to $1/4\varepsilon^2$. Since ε is very small, $1/4\varepsilon^2$ dominates the term L. Next, consider the square root term, which can be approximated as: $\sqrt{\left(1 - \frac{1}{4\varepsilon^2} + \frac{2s}{A}\right)^2 - \frac{4s^2}{A^2}}$. For small ε, the dominant contribution to the expression inside the square root is $(1/4\varepsilon^2)^2$. This allows us to further

approximate the square root as: $\sqrt{\left(\frac{1}{4\varepsilon^2}\right)^2+\cdots}$. Since both $1/4\varepsilon^2$ and $\sqrt{\left(\frac{1}{4\varepsilon^2}\right)^2+\cdots}$ diverge at a same order in ε, these two terms cancel out in the expression for $\widehat{M}_l$, but not in $\widehat{M}_u$ (☐). This suggests that positive complexity-stability relationship does not appear actually if the two types of higher-order interactions occur with a comparable frequencies within the system. Here, when $p = p_{\widehat{M}} = \frac{1}{2} + \frac{1}{2\sqrt{1+\frac{4S}{A}}}$, the two thresholds are equal or $\widehat{M}_l = \widehat{M}_u = \widehat{M}$. Then, when $p > p_{\widehat{M}}$, the conditions (7) becomes $0 < M$. It indicates that the system is always stable and does not show a positive complexity-stability relationship (always stable) if positive higher-order interactions are sufficiently numerous. On the contrary, when $p < p_{\widehat{M}}$, the system can show positive complexity-stability relationship. The positive complexity-stability relationship is likely to appear as *s*/*A* becomes smaller (Fig. S4).

References

1. May, R. M. (1972). Will a large complex system be stable?. *Nature*, 238, 413-414.
2. Allesina, S., & Tang, S. (2015). The stability–complexity relationship at age 40: a random matrix perspective. *Population Ecology*, 57, 63-75.

SI Figures

Fig. S1. Effects of connectance on complexity-stability relationship in different types of network with varying the proportion of positive HOI (p). (a) Food-web. (b) Competition web. (c) Mutualistic web. In the upper and lower panels, C = 0.2 and 0.4, respectively. Black dashed lines are the results without HOIs (h = 0). Solid lines are the results with HOIs (h = 1). Colors in solid lines represent different values of p. The colors in the lower panels correspond to the same parameter values as those indicated in the upper panels. The other information is same as those in Fig. 4 in the main text.

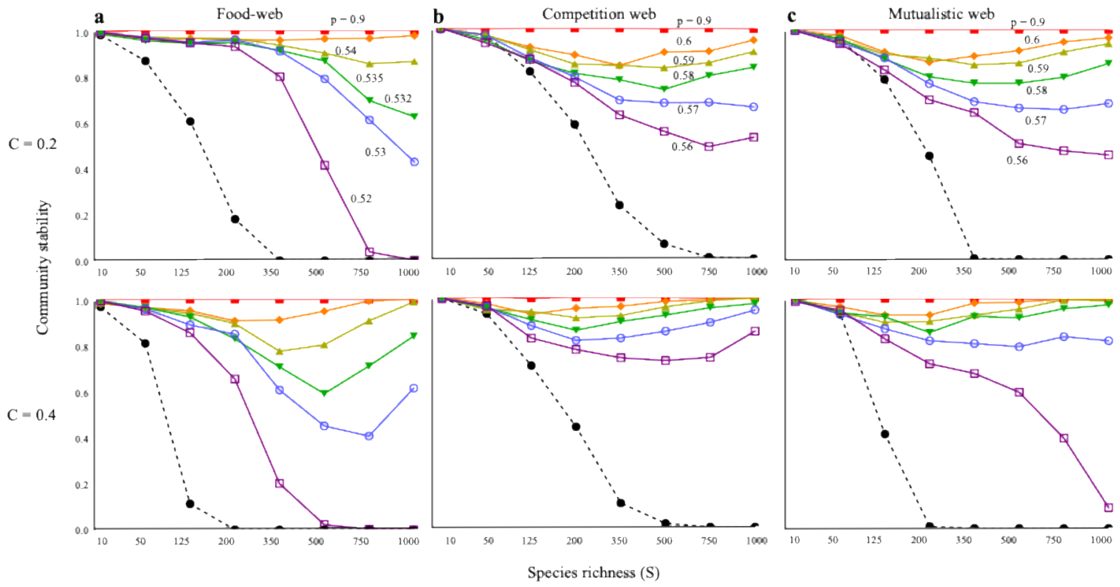

Fig. S2. Complexity-stability relationship in the model assuming independent occurrences of direct and higher-order interactions among species pairs. In this simulation, higher-order interactions can occur independently of direct species interactions (although in the main text, those occur "only" among interacting species pairs). Black dashed lines are the results without HOIs (h = 0). Solid lines are the results with HOIs (h = 1). Colors in solid lines represent different values of p (proportion of positive higher-order interaction). (a-d) has different values of θ (variability). I assume C (connectance) = 0.3.

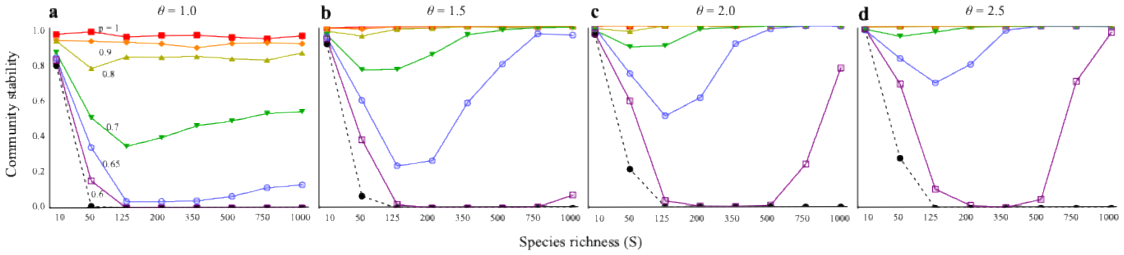

Fig. S3. Effects of the strength of higher-order interactions (h) on a complexity-stability relationship. In the main text, the strengths of higher-order interactions and pairwise direct interactions are assumed to be equal (h = 1 or 0). Here, I relax this assumption to explore how varying the relative strength of higher-order interactions, compared to pairwise interactions, affects the relationship between complexity and stability. I assume C (connectance) = 0.3, θ (variability) = 3 and p (proportion of positive higher-order interaction) = 0.65.

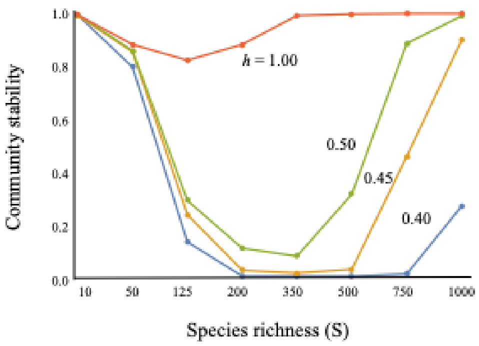

Fig. S4. Effects of HOI on complexity-stability relationship in the mathematical analysis. (a) Change of local stability in spaces of complexity and proportion of positive HOI. (i) Negative complexity-stability (rigorously, it can be reversed to positive complexity-stability in sufficiently high complexity). Note that in an extreme where p approaches to 0.5, the stability is always unstable. (ii) Positive complexity-stability. (iii) Always stable. The ranges of (i) and (ii) changes with increasing complexity, as shown in the upper side of (a). Orange and blue lines are $\widehat{M}_u$ and $\widehat{M}_l$, respectively, in the local stability conditions (7) in Appendix. I assume s (the density-dependent self-regulation of each species) = 0.25 and A (the interaction coefficient between species) = 0.05. (b) Parameter dependence of the stability condition (7) in Appendix. Increasing A broadens the region of positive complexity-stability. I assume s = 0.25.

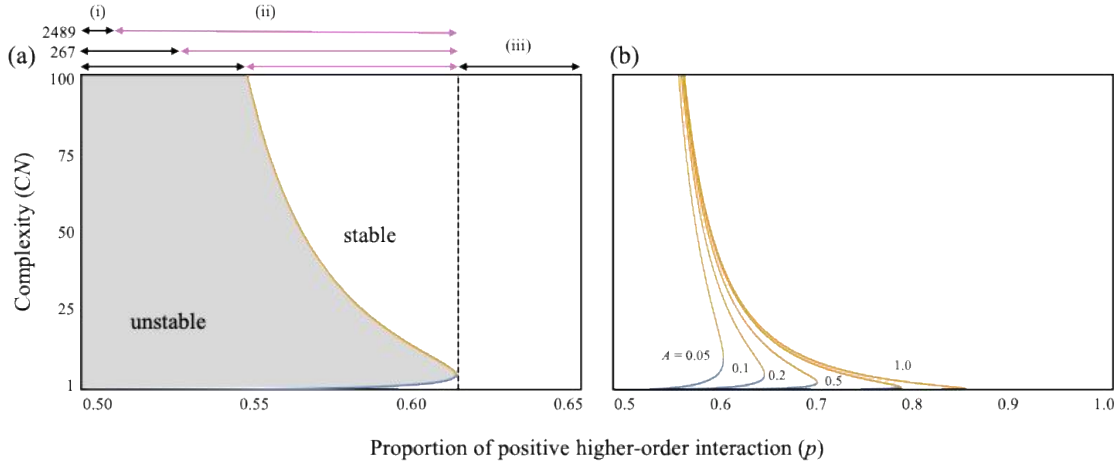

Supplement: Supplementary file 1 — Supplementary Information. [file 41598_2025_15320_MOESM1_ESM.pdf]
